# The double-edged sword of human-AI collaboration on design students’ competence development: The mediating roles of challenge and hindrance appraisals and the asymmetric moderation of teacher AI leadership

**DOI:** 10.1371/journal.pone.0360722

**Published:** 2026-10-16

**Authors:** Xiang Wang, MingXing Li, Jia Du, YU Yao, Jiabei Jiang

**Affiliations:** 1 School of Art and Design, Nanjing Institute of Technology, Nanjing, China; 2 Department of Design, Shanghai Academy of Fine Arts, Shanghai University, Shanghai, China; 3 Design and Craft Graduate School, Hongik University, Seoul, Korea; 4 School of Industrial Design, Nanjing University of the Arts, Nanjing, China; Hunan Normal University, CHINA

## Abstract

The deepening integration of generative AI into design education is driving a paradigm shift in human-computer AI collaboration (HAIC) -from mere tool assistance toward intelligent synergy. However, its dual impact on the competency development of design students and the underlying mechanisms remain insufficiently understood. Grounded in the Theory of Stress Appraisal, this study constructs a research model to examine the double-edged sword effect of HAIC on Design Competence (DC), introducing teacher AI Leadership (AIL) as a moderating variable. The aim is to reveal the psychological mechanisms and boundary conditions through which HAIC influences DC. Employing a questionnaire survey, this study conducted an empirical investigation involving 483 design students in East China. Data analysis was performed using SPSS 24.0 and Amos 23.0, encompassing reliability and validity tests, regression analysis, and Bootstrap mediation analysis. The results indicate that HAIC influences DC through dual pathways: it positively enhances DC by fostering Challenge Appraisal (CA) (β = 0.283, p < 0.01), which in turn positively predicted competence development (β = 0.363, p < 0.01), yielding a significant indirect effect of 0.103 (p < 0.01). Simultaneously, HAIC triggered Hindrance Appraisal (HA) (β = 0.271, p < 0.01), which negatively predicted competence development (β = −0.229, p < 0.01), yielding a significant indirect effect of −0.062 (p < 0.01). This constitutes a partial mediating mechanism characterized by “one positive, one negative” effect. Furthermore, Teacher AIL exhibits an asymmetric moderating effect: it significantly buffers the positive impact of HAIC on HA (β = −0.112, p < 0.01), indicating that high AIL can mitigate cognitive load and anxiety arising from technological uncertainty, yet it does not significantly amplify the positive pathway via CA (β = 0.001, p > 0.05). The findings suggest that the impact of HAIC on DC is not linear but rather a dialectical process contingent upon individual cognitive appraisal. The core value of Teacher AIL lies in “risk mitigation” rather than “creativity stimulation,” offering a theoretical foundation for the intelligent transformation of educational design. At the practical level, it is imperative to facilitate from knowledge deliverers to cognitive facilitators in AI-augmented design education. By systematically developing AI instructional competencies and reforming assessment systems, educators can guide students toward establishing a healthy relationship with HAIC, thereby achieving the synergistic advancement of technological empowerment and humanistic literacy.

## Introduction

The rapid adoption of generative AI is reshaping global design‑education ecosystems and talent‑training paradigms.With the widespread integration of international platforms such as MidJourney, Stable Diffusion, and DALL-E, alongside the rapid advancement of domestic tools like ZCOOL Design Agent and Instant Design AI, AI has transcended its role as a mere auxiliary “experimental tool” to become an “intelligent collaborator” deeply embedded in the core workflows of creative conceptualization, scheme iteration, and decision-making [1,2].

Against this macro-context, design education in China is undergoing a structural transformation. Since the promulgation of the Development Plan for the Next Generation of Artificial Intelligence, over 600 universities nationwide have been authorized to establish undergraduate programs in AI. By 2025, more than 70 institutions are projected to launch interdisciplinary tracks such as “Computational Design” and “AI and Creative Design.” These developments signify that AI-integrated design education has entered a phase of large-scale implementation, shifting educational objectives from simple “tool proficiency” to fostering a multidisciplinary design mindset centered on HAIC [3].

Existing scholarship highlights the multidimensional empowerment potential of AI in design education [4]. Firstly, regarding creative expansion, generative AI breaks through the constraints of individual experience and conventional techniques, capable of generating a vast array of visual proposals in short timeframes, thereby serving as a rich source of inspiration for design concepts [5,6]. Empirical research by Doshi and Hauser [7] demonstrates that exposure to AI-generated stimuli significantly enhances the novelty of work produced by highly creative individuals. Secondly, concerning learning efficacy, AI substantially lowers the technical threshold for visual expression, enabling beginners to rapidly produce professional-grade outcomes and effectively flattening the learning curve for foundational skills [8]. He and Zhang [9] further confirm that AI-enhanced educational support systems markedly improve both creative output quality and learner engagement. Thirdly, at the pedagogical paradigm level, technology is driving a shift from traditional “unidirectional instruction” to “personalized collaboration,” thereby restructuring the underlying logic of innovation and entrepreneurship education in higher education [10]. Drawing on the affordances of AIGC, Min et al. [11] have explored novel pathways for cultivating students’ interdisciplinary thinking and cross-media competencies, charting a new course for innovative talent cultivation in architectural education at Tsinghua University.

However, the double-edged sword effect of AI technology cannot be ignored. While AI yields substantial efficiency gains, it concurrently engenders profound concerns regarding cognitive impairment, creative convergence, ethical dilemmas, and emotional detachment.

First, cognitive offloading may undermine deep learning capabilities [12]. Risko and Gilbert [13] posit that although delegating cognitive tasks to external tools alleviates immediate cognitive load, it risks precipitating a “use it or lose it” atrophy of endogenous cognitive faculties. Corroborating this, a meta-analysis by Zhai et al. [14] cautions that over-reliance on AI-driven “cognitive shortcuts” may induce a deterioration in students’ critical thinking skills.

Second, algorithmic bias exacerbates creative homogenization and design fixation [15]. A controlled experiment conducted by Wadinambiarachchi et al. [16] at CHI 2024 revealed that AI-assisted cohorts exhibited significantly stronger design fixation effects compared to their unaided counterparts, manifesting as markedly diminished diversity and originality in proposals. This phenomenon is primarily attributable to the misalignment between training data and real-world exigencies, coupled with students’ uncritical acceptance of AI outputs.

Furthermore, ambiguities surrounding originality and academic integrity pose escalating challenges. Due to nebulous copyright frameworks, students display considerable variance in adjudicating the originality of AI-generated content [17], thereby compelling higher education institutions to urgently redefine the contours of “originality” and its evaluative metrics [18].

Finally, the erosion of emotional competence and the entrenchment of socio-cognitive biases are becoming increasingly salient. Research by Zheng et al. [19] indicates that students often forfeit independent judgment by uncritically acquiescing to the outputs of Large Language Models; such path dependency ultimately culminates in the ossification of cognitive biases.

In summary, prior scholarship has separately documented both benefits and risks of generative AI within design education, yet three important empirical gaps remain. First, few empirical studies have simultaneously integrated both positive and negative consequences of human‑AI collaboration within a single unified model to test its double‑edged‑sword mechanism. Second, existing work largely focuses on student‑level boundary factors such as AI literacy, with limited empirical attention paid to teacher‑level AI leadership as a contextual moderator that may mitigate or amplify AI‑related outcomes. Third, the micro‑level psychological processes that translate HAIC experiences into design‑competence gains or losses remain insufficiently tested; prior work rarely distinguishes between challenge‑related versus hindrance‑related cognitive appraisals as parallel mediating pathways.

In light of this, drawing upon Stress Appraisal Theory, this study constructs a theoretical model to elucidate the double-edged sword effect of HAIC on design students’ DC. It innovatively introduces Teacher AIL as a key moderating variable. This research aims to demystify the underlying mechanisms through which AI influences design education, thereby providing systematic theoretical referents and actionable insights for the intelligent transformation of design pedagogy.

## Literature review

### Research on human-AI collaboration

As a core paradigm within human–robot interaction (HRC), human–robot collaboration was originally defined by ISO (2011) [20] as an operating mode in which humans and robots share the same workspace. Its core principles role division, task delegation, and mutual adaptation,were initially developed for physical manufacturing, where humans perform dexterous manipulation and high-level decision-making while robots undertake repetitive, high-intensity, or high-precision operations [21,22]. This division manifests through two mechanisms: physical collaboration, mediated by force and torque sensing with real-time intent prediction, and non-contact collaboration, which relies on voice, gesture, and implicit-intention inference [21].

Although HRC originally described physical human‑robot interaction in manufacturing settings, its core logic of role division, task allocation and shared cognition can be analogically extended to cognitive knowledge‑work scenarios with generative AI. In design education, HAIC is a cognition‑centered collaboration rather than physical manipulation‑centered collaboration.

As intelligent technologies increasingly exhibit human-like attributes autonomous learning, affective feedback, and even ethical judgment the role of AI in the workplace is shifting from passive instrument to proactive “team member.” Specifically, HAIC refers to a model in which humans and AI jointly accomplish complex tasks through continuous, iterative interactions within a shared workspace and a unified dynamic system [23].

In the context of design education, the hybrid workforce model enabled by human–AI collaboration has emerged as the dominant form of design innovation [24]. This symbiotic relationship manifests as mutual empowerment: on the one hand, students drive the intelligent evolution of AI systems by articulating design requirements, contributing tacit knowledge, and generating behavioral data; on the other hand, by assuming routine and repetitive design tasks, AI liberates students’ cognitive resources, enabling them to engage in more creative and high-value exploration, thereby stimulating DC. Grounded in this mutually beneficial human–AI symbiosis, this study aims to systematically investigate the impact of HAIC on students’ DC and the underlying mechanisms involved.

### Theory of stress appraisal

The theory of stress appraisal, pioneered by Lazarus and Folkman [25], asserts that cognitive evaluations of stressors constitute the principal mechanism governing emotional responses and coping behaviors. The theory delineates a dual-stage appraisal process, wherein primary appraisal assesses the relevance of environmental demands to personal goals, bifurcating into CA and HA [26]. CA is characterized by a perceived potential for mastery, gain, or personal growth, predicated on the expectancy that effort will result in commensurate rewards [25,27]. In contrast, HA reflects a perception that effort–reward contingencies are dissociated, fostering the belief that the stressor functions as an obstruction to well-being and development [27].

Empirical evidence suggests that this appraisal mechanism extends beyond traditional stressors to technologically mediated environments. Notably, Jing and Wei [28] reveal that AI-induced anxiety can differentially shape innovative behaviors among new-generation employees, eliciting facilitative effects via work restructuring or inhibitory effects via defensive avoidance.

Integrating this theoretical lens with the HAIC framework, this study posits that students’ perceptions of human–AI collaboration manifest a parallel dichotomy: CA (construing AI as a catalytic asset for augmenting DC) versus HA (construing AI as a vocational threat). Consequently, this study operationalizes CA and HA as pivotal psychological mediators, aiming to elucidate the mechanistic pathways through which stress and stress perception influence DC.

### Design competence

DC is a multidimensional and composite construct, typically defined in academia as an individual’s or team’s capacity to integrate abstract design knowledge, cognitive strategies, and operational skills through a systematic iterative process to resolve complex problems or generate novel value [29,30]. This competency generally encompasses five core dimensions: technical skills [31], creative ability [32], critical thinking [33], aesthetic literacy [34], and humanistic literacy [35], each elaborated as follows:(1) Technical Skills: The foundational operational and execution capabilities that enable design students to materialize their design intentions via design software, AI-assisted tools, and traditional media (e.g., hand-drawing).(2) Creative Ability: Serving as the core engine of DC, this refers to the capacity to produce design solutions that are novel, distinctive, and pragmatically effective.(3) Critical Thinking: The cognitive capacity to conduct rigorous analysis, rational evaluation, value judgment, and decision optimization throughout the design lifecycle. (4) Aesthetic Literacy: A form of comprehensive cultivation accrued through sustained aesthetic practice, encompassing perceptual sensitivity, stylistic recognition, interpretive command of formal languages, and the appreciation of design quality.(5) Humanistic Literacy: Constituting the axiological foundation of DC, this dimension reflects an individual’s capacity to perceive and empathize with human culture, social ethics, and authentic human needs.

### AI leadership

Digital leadership emphasizes a leader’s ability to use digital technologies to drive organizational change and foster innovation [36]. The surge in generative AI has further expanded the scope of leadership, giving rise to the new concept of AIL [37].

In the field of higher education, related research is rapidly emerging. Dunnigan et al. [38] point out that school administrators’ strategic choices and response models regarding generative AI directly determine teachers’ willingness to adopt it and students’ usage behaviors. Khairullah et al. [39] emphasize that universities urgently need to establish responsible AI strategic leadership to drive sustainable development by deeply aligning AI integration goals with institutional missions.

In the context of the generative AI era, the core objective of AIL in the educational setting is to guide students in learning to use AI, progressing to effectively leveraging AI for learning, and ultimately achieving collaborative innovation with AI. This requires AI educators to move beyond mere technical application and instead view AI as a creative partner, guiding students to think symbiotically with AI and engage in deep interdisciplinary integration and innovation [40]. Within the present study, perceived teacher AIL is defined as students’ subjective perceptions of educators’ comprehensive capacity to deploy AI technology to optimize instructional design, facilitate educational transformation, and support students to build healthy HAIC relationships. Importantly, AIL is measured from the student perspective via self‑report perceptions of teacher behaviors; it is not an objective trait‑based assessment of teachers’ actual leadership capabilities.

## Theoretical framework and research hypotheses

### Double-edged sword effect hypothesis

Extant literature suggests that awareness of AI’s impact can trigger two divergent behavioral responses among employees: creative use and avoidance [41]. Concurrently, drawing on Stress Appraisal Theory, AI-assisted intelligence has been shown to exert a significant“double-edged sword”effect on employees’innovative behavior [42]. Grounded in this theoretical lens, the present study posits that within emerging AI contexts, distinct dimensions of HAIC activate differentiated psychological appraisal pathways.

First, HAIC provides students with robust cognitive support and tool empowerment, significantly enhancing their sense of control and self-efficacy in design tasks [43]. This beneficial effect encourages students to construe collaborative scenarios as opportunities for personal growth, thereby reinforcing their CA.

Second, HAIC also generates novel learning demands. Specifically, these manifest as challenge demands characterized by the high cognitive effort required to master complex tools, the mental load of prompt engineering, and the pressure for continuous upskilling driven by technological iteration [44]. Conversely, they also constitute hindrance demands, encompassing threatening factors such as uncertainty regarding AI output quality, copyright disputes, and ethical risks [45]. Once these cumulative demands exceed students’ current competence thresholds, they precipitate anxiety over the potential loss of design autonomy, subsequently triggering HA.

Based on this theoretical reasoning, this study proposes the following hypotheses:

H1a: HAIC positively influences individuals’ CA.
H1b: HAIC positively influences individuals’ HA.

Grounded in Stress Appraisal Theory, extant research indicates that AI adoption exerts a dual impact on innovative behavior. Dong et al. [46] demonstrate that AI can foster innovation by enhancing CA, yet may simultaneously inhibit it by exacerbating HA. Drawing on a sample of primary and secondary school teachers, Kong et al. [47]substantiated the ambivalent (“double-edged sword”) nature of AI adoption. Their findings delineate that while challenge appraisal exhibits a significant positive association with innovative behavior, threat appraisal demonstrates a contrasting negative relationship. Extending this logic to design education, this study posits that CA and HA function as distinct psychological pathways through which HAIC influences DC.

Specifically, CA operates as an approach motivation, eliciting positive affective experiences. When students perceive HAIC as a manageable challenge, they are more inclined to engage in logical deconstruction and critical reconstruction of AI outputs, thereby enhancing their DC. Conversely, HA activates avoidance motivation, leading to anxiety and burnout. If students perceive AI as a threat to originality or a source of cognitive overload, their sense of autonomy becomes compromised. Consequently, they may resort to defensive reliance on AI to circumvent deep cognitive engagement, resulting in the atrophy of independent DC due to the “use it or lose it” principle.

Based on this reasoning, the following hypotheses are proposed:

H2a: CA positively influences design students’ DC.
H2b: HA negatively influences design students’ DC.

Furthermore, this study argues that stress perception serves as a pivotal mediating mechanism linking HAIC and DC. These two pathways operate in parallel yet in opposing directions, collectively accounting for the paradoxical nature of HAIC’s impact. Therefore, the following mediating hypotheses are proposed:

H3a: CA positively mediates the relationship between HAIC and DC.
H3b: HA negatively mediates the relationship between HAIC and DC.

### Moderation effect hypotheses

Extant research has widely corroborated that leadership serves as a pivotal boundary condition shaping individual and organizational behavior. For instance, Zhu et al. [48] demonstrated that digital leadership positively moderates the effect of perceived technological shock on craftsmanship; Zhang and Zheng [49] revealed that crisis leadership can buffer the adverse impact of crisis intensity on employees’ proactive behavior; and Fousiani et al. [50] validated the moderating role of leader power in the relationship between competitive climate and AI acceptance. Grounded in this scholarly consensus, this study introduces AIL as a critical moderator to examine its role in mitigating or amplifying the “double-edged sword” effect of HAIC. Specifically:

On the gain pathway, instructors with high AIL are proficient in identifying and integrating technological resources, guiding students to leverage AI-assisted learning efficiently. Such guidance reinforces students’ CA toward job resources, thereby accelerating the transformation of learning resources into DC. Accordingly, AIL is expected to strengthen the positive impact of HAIC on CA, consequently enhancing DC.

On the loss pathway, instructors with high AIL can effectively alleviate students’ helplessness and cognitive load when confronting AI complexities through timely support and structured scaffolding. This intervention mechanism prevents task demands from escalating into HA, thereby mitigating the negative depletion effect. Hence, AIL is anticipated to suppress the positive relationship between HAIC and HA (i.e., a buffering effect), ultimately safeguarding DC.

#### Based on the above analysis, the following hypotheses are proposed.

H4a: AIL positively moderates the strength of the HAIC → CA path.
H4b: AIL negatively moderates the strength of the HAIC → HA path.
H5a: AIL positively and indirectly moderates the strength of the HAIC → CA → DC path.
H5b: AIL negatively and indirectly moderates the strength of the HAIC → HA → DC path.

### Theoretical model

Based on the above literature review and theoretical analysis, this study combines the expanded theory of stress appraisal to construct a moderated dual‑mediation theoretical framework explaining the influence of HAIC on design students’ DC (as shown in Fig 1).Conceptual model depicting the hypothesized relationships among HAIC, CA, HA, perceived teacher AIL, and DC. H1a‑H5b denote the corresponding research hypotheses.

**Fig 1:**
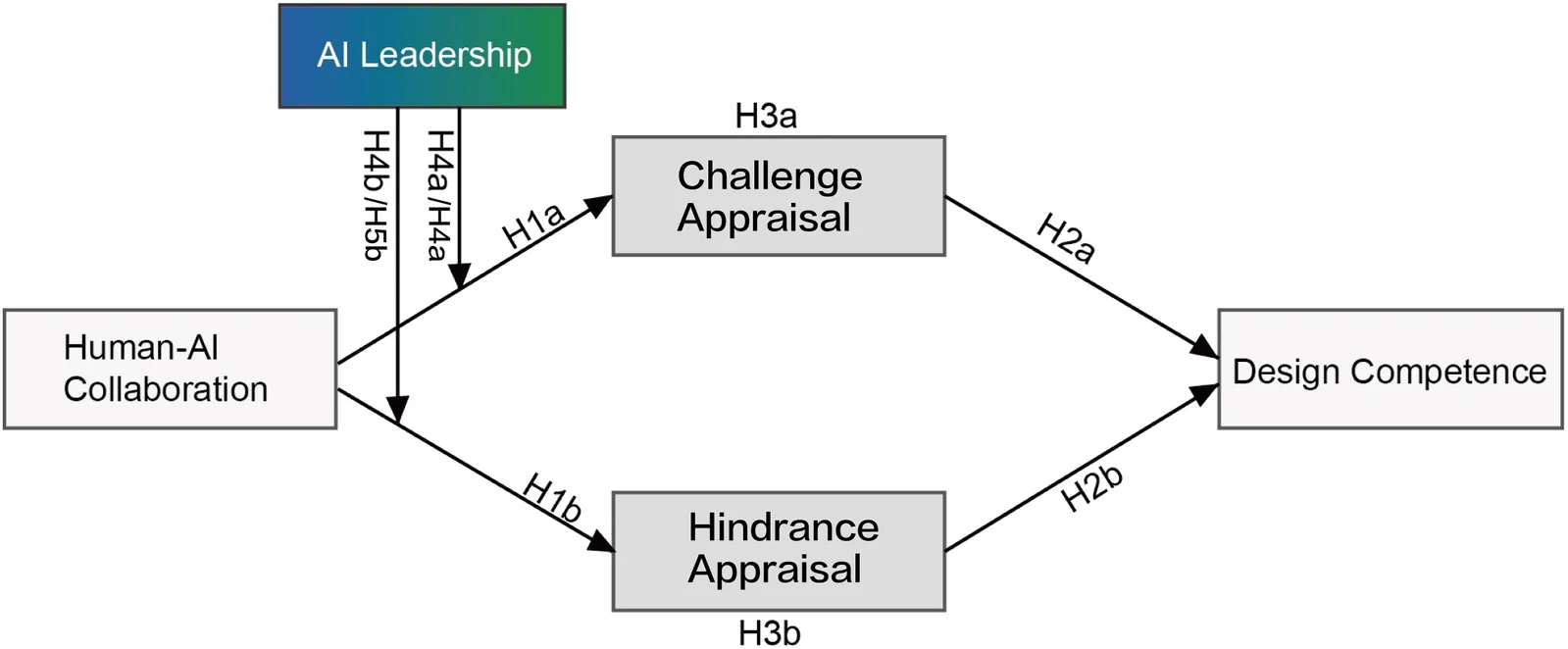
Research framework of the moderated dual‑mediation model.

## Materials and methods

### Definition and measurement of variables

This study constructs a research model comprising five core latent variables: HAIC, CA, HA, AIL, and DC. These variables collectively form a theoretical framework aimed at elucidating the mechanisms through which human-AI interaction influences the development of design students’ professional competencies.

To ensure the content validity and reliability of the measurement instruments, the operational definitions and measurement items for each latent variable were rigorously developed through an extensive review of existing literature and theoretical frameworks. The specific measurement indicators for each variable are presented in detail in Table 1.

**Table 1:** Definitions of variable operability and reference scales.

Variables	Definition of operability	Measurement items	References
HAIC	The interactive process in which design students collaborate with AI tools and systems through division of labor, command interaction, and joint decision-making to complete design tasks, solve problems, and optimize solutions within their academic and practical activities.	HAIC1: I actively collaborate with AI to complete design tasks.	[51–54]
		HAIC2: I frequently use AI tools to assist my design learning and practice.	
		HAIC3: I involve AI in the process of identifying and evaluating design works.	
		HAIC4: I appropriately select and utilize different AI tools based on specific design task requirements.	
		HAIC5: I am proficient at instructing AI to execute specific design modifications.	
		HAIC6: AI is deeply involved in my design problem-solving process.	
		HAIC7: AI plays a supportive role in my design decision-making process.	
CA	The cognitive appraisal whereby design students perceive the task requirements and technical challenges inherent in HAIC as opportunities for DC growth and professional breakthroughs, thereby generating motivation for proactive engagement.	CA1: Collaborating with AI provides me with opportunities to learn new design skills and knowledge.	[25,42,46,55]
		CA2: I view addressing AI-generated suggestions and critiques as a valuable challenge.	
		CA3: Collaborating with AI helps me overcome existing bottlenecks in my design process.	
		CA4: I regard AI-assisted design as a vital opportunity to enhance my professional capabilities.	
		CA5: I feel motivated when confronted with complex design tasks involving AI.	
HA	The cognitive appraisal whereby design students perceive the technical uncertainties, cognitive load, and ethical risks inherent in HAIC as stressors that threaten their design autonomy, reduce efficiency, or hinder professional development, leading to negative coping tendencies.	HA1: I feel that AI sometimes restricts my creative expression and independent thinking.	[25,42,46,55]
		HA2: Understanding and debugging AI outputs imposes significant pressure on me.	
		HA3:AI-generated proposals often require extensive rework, which reduces my design efficiency.	
		HA4: I am concerned that over-reliance on AI will lead to the deterioration of my fundamental design skills.	
		HA5: I often feel anxious, uncertain, or a lack of control during AI-assisted design processes.	
AIL	The comprehensive competency of design educators to utilize AI technology to optimize instructional design, guide students in the appropriate use of AI tools, foster critical evaluation of AI-generated content, and support students in establishing healthy HAIC relationships.	AIL1: Instructors integrate AI competency mastery into course learning objectives and teaching activities.	[40,56–58]
		AIL2: Instructors are skilled at designing human-AI collaborative tasks that leverage the respective strengths of both parties.	
		AIL3: Instructors provide personalized feedback and guidance tailored to my specific challenges in applying AI.	
		AIL4: Instructors guide students to understand the underlying logic of AI outputs (e.g., data bias, probabilistic mechanisms) rather than focusing solely on results.	
		AIL5: Instructors provide necessary resources and technical support for AI-assisted design learning.	
		AIL6: Instructors guide us in critically examining copyright and ethical risks associated with AI-generated content.	
DC	The comprehensive ability of design students to integrate technical skills, creative abilities, critical thinking, aesthetic literacy, and humanistic literacy to solve complex problems and create design value through systematic design processes.	DC1: I am proficient in using design software and AI tools to execute the design process from concept to implementation.	[29,32,33,35]
		DC2: I can think divergently and propose design solutions that are both novel and practical.	
		DC3: I can systematically analyze design problems and propose targeted optimization strategies.	
		DC4: I can critically evaluate the innovation and aesthetic quality of design expressions.	
		DC5: I can identify user needs and social values and integrate them into my design proposals.	

### Questionnaire design and data collection

This study employed a questionnaire survey to collect empirical data. To ensure content validity and structural rigor, we strictly adhered to the pilot testing protocols proposed by Oksenberg and Kalton [59] and conducted a small-scale pre-survey in Jiangsu Province from December 9–15, 2025. A total of 80 questionnaires were distributed, yielding 68 valid responses after data cleaning, which satisfies the recommended sample size range of 25–75 for pilot studies. Based on the pilot data, items with insufficient discriminant validity were removed through reliability analysis, item analysis, and unidimensionality tests, thereby refining and finalizing the questionnaire.

The final questionnaire consisted of non‑scoring introductory text, followed by 35 scored items. Among them, seven items captured demographic background (gender, age, educational attainment, major discipline, AI‑tool‑usage experience, AI‑usage frequency, and prior AI‑design coursework) using categorical response formats. The remaining 28 items were seven‑point Likert‑scale items measuring the five core latent constructs (HAIC, CA, HA, AIL, DC), ranging from 1 (strongly disagree) to 7 (strongly agree).

This study was approved by the Institutional Review Board of the School of Art and Design at Nanjing Institute of Technology (Approval No. NJIT-IRB-20251115–01), and all participants provided informed consent. The formal survey was administered via the Wenjuanxing platform from March 12–28, 2026, targeting college students in East China (Jiangsu, Zhejiang, and Shanghai) using convenience sampling. A total of 602 responses were collected; after excluding invalid samples, 483 valid questionnaires were retained, resulting in a valid response rate of 80.23%. Given the planned use of Maximum Likelihood (ML) estimation for Structural Equation Modeling (SEM), the sample size (N = 483) corresponds to an item-to-sample ratio of approximately 1:17.25, significantly exceeding the 1:10 threshold recommended by Jackson [60], thus confirming the adequacy of the sample size for robust statistical inference. Data analysis was performed using SPSS 24.0 and Amos 23.0.

## Results

### Descriptive statistics

The final sample comprised 483 valid participants. In terms of demographic characteristics, female respondents accounted for 59.21% (n = 286), while males represented 40.79% (n = 197). The age distribution was predominantly concentrated among individuals aged 22–24 (42.86%) and 19–21 (35.82%). Regarding educational background, undergraduates constituted the majority (77.64%), with academic majors spanning Visual Communication Design (24.64%), Product Design/Industrial Design (22.15%), Environmental Design/Architecture (20.08%), Digital Media Art (16.56%), and Fashion Design and other related fields (16.56%).

Concerning AI tool utilization, 53.42% of participants reported having used AI for one year or more. Furthermore, a significant majority (75.98%) employed AI tools in over 30% of their coursework, with 49.07% utilizing AI in 30%–70% of design tasks and 26.92% relying on AI for more than 70% of their assignments. Notably, despite the high penetration rate of AI applications within design practice, only 18.84% (n = 91) of respondents indicated having systematically undertaken AI-related design courses. This disparity suggests that specialized AI instruction has yet to become a mainstream component of contemporary design education curricula (As shown in Table 2).

**Table 2:** Frequency distribution of sample demographic characteristics and AI‑usage profiles.

Variable	Category	Frequency	Percentage (%)
Gender	Male	197	40.79
	Female	286	59.21
Age	18 and under	20	4.14
	19–21	173	35.82
	22–24	207	42.86
	25 and older	83	17.18
Education	Bachelor’s degree or below	375	77.64
	Master’s degree	79	16.36
	Doctoral degree	29	6.00
Major	Visual Communication Design	119	24.64
	Product Design / Industrial Design	107	22.15
	Environmental Design / Architecture	97	20.08
	Digital Media Art	80	16.56
	Fashion Design & Others	80	16.56
Experience	< 3 months	62	12.84
	3–6 months	86	17.81
	6–12 months	77	15.94
	≥ 1 year	258	53.42
Frequency of AI Use	Occasional (< 30% of tasks)	116	24.02
	Frequent (30%–70% of tasks)	237	49.07
	Primary (> 70% of tasks)	130	26.92
AI Design Courses Taken	No	392	81.16
	Yes	91	18.84
Total		483	100.00

### Reliability analysis

Reliability analysis, fundamentally regarded as an assessment of internal consistency, evaluates the stability and dependability of measurement outcomes. To safeguard the rigor of subsequent statistical procedures, it is imperative to verify the reliability of valid questionnaire data prior to conducting further analysis. Within the social sciences, Cronbach’s α coefficient serves as the predominant metric for this purpose. Conventional interpretation criteria suggest that a coefficient exceeding 0.9 reflects exemplary reliability, 0.8–0.9 indicates high reliability, 0.7–0.8 denotes acceptable reliability, and values below 0.6 signal a need for substantial revision.

As evidenced in Table 3, the reliability coefficients across all dimensions surpassed the 0.8 threshold, demonstrating a high level of internal consistency within the research data. Furthermore, an examination of the “Cronbach’s α if Item Deleted” column revealed no significant potential for coefficient improvement through item removal; thus, retaining all items is justified. Concurrently, the Corrected Item-Total Correlation (CITC) values for all items exceeded 0.4, signifying robust inter-item correlations and reinforcing the instrument’s reliability. In summary, the collective findings confirm that the dataset exhibits high reliability, rendering it suitable for advanced analytical techniques.

**Table 3:** Cronbach’s alpha reliability analysis.

Variable	Item	CITC	α Coefficient for Deleted Item	Cronbach’s α coefficient
HAIC	HAIC1	0.757	0.906	0.919
	HAIC2	0.738	0.908	
	HAIC3	0.739	0.908	
	HAIC4	0.750	0.907	
	HAIC5	0.746	0.907	
	HAIC6	0.757	0.906	
	HAIC7	0.759	0.906	
CA	CA1	0.723	0.845	0.876
	CA2	0.684	0.855	
	CA3	0.737	0.842	
	CA4	0.689	0.854	
	CA5	0.694	0.852	
HA	HA1	0.714	0.857	0.882
	HA2	0.708	0.858	
	HA3	0.727	0.854	
	HA4	0.727	0.854	
	HA5	0.706	0.859	
AIL	AIL1	0.728	0.887	0.903
	AIL2	0.733	0.887	
	AIL3	0.739	0.886	
	AIL4	0.728	0.887	
	AIL5	0.735	0.886	
	AIL6	0.748	0.884	
DC	DC1	0.696	0.848	0.874
	DC2	0.708	0.846	
	DC3	0.666	0.856	
	DC4	0.730	0.840	
	DC5	0.708	0.845	

### Exploratory factor analysis

#### KMO and Bartlett’s test.

Validity denotes the degree to which an instrument precisely captures the targeted psychological and behavioral constructs, reflecting the accuracy and dependability of the measurements. In assessing the suitability for factor analysis, the Kaiser-Meyer-Olkin (KMO) measure evaluates sampling adequacy by comparing the magnitudes of observed correlation coefficients to partial correlation coefficients, ranging from 0 to 1. Conventionally, a KMO value exceeding 0.7 signifies adequate suitability, while values above 0.9 indicate exceptional appropriateness. Additionally, Bartlett’s Test of Sphericity examines the null hypothesis that the correlation matrix is an identity matrix. A significance level (p < 0.05) rejects this hypothesis, confirming the presence of substantial inter-item correlations. As demonstrated in Table 4, the KMO statistic of 0.887 and the significant Bartlett’s test result (p < 0.05) collectively indicate that the dataset possesses high sampling adequacy and strong inter-variable relationships, thereby justifying the use of factor analysis and suggesting robust construct validity.

**Table 4:** KMO and Bartlett’s test.

KMO value		0.887
Bartlett Sphericity Test	Approximate Chi-Square	7811.563
	df	378
	*p*-value	<0.001

### Variance contribution rate

Using the system’s default method to extract factors with eigenvalues greater than 1, principal component analysis was performed on the 28 items. The results yielded five common factors, which accounted for 67.526% of the variance, a relatively satisfactory outcome (as shown in Table 5). Common method bias was tested using Harman’s single-factor method. The results showed that the maximum explanatory power of the principal component in the principal component analysis was 23.376%, which is far below the 40% cutoff criterion, These results suggest that common‑method variance is unlikely to pose a serious threat to the interpretation of the present findings.

**Table 5:** Total variance explained.

Factor number	Initial Eigenvalues			Extraction Sums of Squared Loadings			Rotation Sums of Squared Loadings		
	Total	% of Variance	Cumulative %	Total	% of Variance	Cumulative %	Total	% of Variance	Cumulative %
1	6.545	23.376	23.376	6.545	23.376	23.376	4.727	16.881	16.881
2	4.482	16.009	39.385	4.482	16.009	39.385	4.069	14.533	31.414
3	3.922	14.006	53.391	3.922	14.006	53.391	3.402	12.15	43.564
4	2.243	8.01	61.401	2.243	8.01	61.401	3.382	12.08	55.644
5	1.715	6.125	67.526	1.715	6.125	67.526	3.327	11.883	67.526
6	0.687	2.454	69.981	–	–	–	–	–	–
7	0.643	2.295	72.275	–	–	–	–	–	–
8	0.584	2.085	74.36	–	–	–	–	–	–
9	0.542	1.937	76.297	–	–	–	–	–	–
10	0.536	1.915	78.213	–	–	–	–	–	–
11	0.497	1.774	79.987	–	–	–	–	–	–
12	0.483	1.726	81.712	–	–	–	–	–	–
13	0.44	1.571	83.283	–	–	–	–	–	–
14	0.433	1.545	84.828	–	–	–	–	–	–
15	0.412	1.472	86.3	–	–	–	–	–	–
16	0.382	1.365	87.665	–	–	–	–	–	–
17	0.377	1.348	89.013	–	–	–	–	–	–
18	0.346	1.237	90.251	–	–	–	–	–	–
19	0.333	1.188	91.438	–	–	–	–	–	–
20	0.322	1.149	92.587	–	–	–	–	–	–
21	0.309	1.105	93.692	–	–	–	–	–	–
22	0.292	1.043	94.735	–	–	–	–	–	–
23	0.274	0.98	95.715	–	–	–	–	–	–
24	0.259	0.926	96.64	–	–	–	–	–	–
25	0.258	0.92	97.561	–	–	–	–	–	–
26	0.25	0.894	98.454	–	–	–	–	–	–
27	0.229	0.818	99.272	–	–	–	–	–	–
28	0.204	0.728	100	–	–	–	–	–	–

The data in this study were rotated using the maximum variance rotation method to identify the correspondences between factors and study items. As shown in Table 6, the absolute values of the factor loadings for all study items are greater than 0.5, indicating that these items are correlated with the factors.

**Table 6:** Table of factor load coefficients after rotation.

Name	Factor Load Coefficient					Commonality
	Factor 1	Factor 2	Factor 3	Factor 4	Factor 5	
HAIC1	0.816	−0.020	0.083	0.051	0.125	0.691
HAIC2	0.786	−0.001	0.117	0.076	0.149	0.660
HAIC3	0.774	−0.068	0.160	0.076	0.167	0.663
HAIC4	0.812	−0.018	0.056	0.113	0.077	0.682
HAIC5	0.779	−0.049	0.115	0.203	0.097	0.673
HAIC6	0.805	−0.022	0.102	0.108	0.116	0.684
HAIC7	0.816	−0.018	0.112	0.109	0.060	0.694
CA1	0.135	0.018	−0.007	0.808	0.153	0.694
CA2	0.148	0.050	−0.022	0.771	0.156	0.644
CA3	0.108	−0.033	0.014	0.804	0.220	0.708
CA4	0.100	0.015	0.001	0.776	0.179	0.645
CA5	0.125	0.012	−0.002	0.784	0.152	0.654
HA1	0.129	0.086	0.809	0.053	−0.044	0.683
HA2	0.114	0.114	0.786	−0.008	−0.150	0.667
HA3	0.152	−0.009	0.818	−0.016	−0.075	0.698
HA4	0.125	0.081	0.811	−0.032	−0.095	0.691
HA5	0.108	0.040	0.815	−0.016	−0.018	0.679
AIL1	−0.042	0.809	0.070	0.037	−0.062	0.666
AIL2	−0.017	0.818	0.054	0.042	−0.014	0.675
AIL3	−0.051	0.820	0.058	−0.017	0.006	0.679
AIL4	−0.024	0.817	0.038	−0.001	0.007	0.669
AIL5	−0.056	0.815	0.068	0.060	0.000	0.675
AIL6	0.026	0.835	0.025	−0.059	0.040	0.703
DC1	0.138	−0.007	−0.095	0.166	0.774	0.654
DC2	0.148	−0.031	−0.066	0.123	0.803	0.688
DC3	0.139	0.013	−0.069	0.221	0.736	0.615
DC4	0.149	0.041	−0.080	0.174	0.801	0.703
DC5	0.121	−0.037	−0.087	0.205	0.778	0.670

### Confirmatory factor analysis

#### Discriminant validity.

This study tested the validity of five variables: HAIC, CA, HA, AIL, and DC. Regarding discriminant validity, confirmatory factor analysis was conducted using AMOS 23.0. The five-factor model exhibited good fit (χ²/df = 2.028, IFI = 0.954, TLI = 0.949, CFI = 0.954, RMSEA = 0.046) and was significantly superior to all competing models, indicating that the five latent variables possess good discriminant validity (as shown in Table 7). Additionally, a confirmatory factor analysis incorporating a common method factor was conducted.

**Table 7:** Results of confirmatory validation analysis.

Model	χ^2^	df	χ^2^/df	IFI	TLI	CFI	RMSEA
Five factor model (HAIC,CA,HA,AIL,DC)	689.639	340	2.028	0.954	0.949	0.954	0.046
Four factor model (HAIC+CA,HA,AIL,DC)	1734.632	344	5.043	0.818	0.799	0.817	0.092
Three factor model (HAIC+CA + HA,AIL,DC)	2866.367	347	8.260	0.670	0.639	0.669	0.123
Two factor model (HAIC+CA + HA + AIL,DC)	4455.166	349	12.766	0.462	0.415	0.460	0.156
Single factor model (HAIC+CA + HA + AIL + DC)	5387.068	350	15.392	0.340	0.284	0.337	0.173

Note: the same below Due to space limitations, only the model with the best fit is displayed for each factor model.

The results indicated that compared to the six-factor model, the model fit did not improve significantly (χ²/df = 2.030, RMSEA = 0.046, CFI = 0.954, TLI = 0.949; ΔRMSEA, ΔCFI, and ΔTLI were all less than 0.02). Taken together, these diagnostics indicate that common‑method bias is unlikely to substantially distort the study’s substantive results.

#### Convergent validity.

In terms of convergent validity, the standardized factor loadings for each variable ranged from 0.724 to 0.806, and the AVE ranged from 0.582 to 0.619. Both met commonly accepted criteria, indicating that the variables possess good convergent validity. In summary, the measurement model in this study demonstrates good reliability and validity and can be used for subsequent analyses (as shown in Table 8).

**Table 8:** Results of reliability and convergent validity tests for each variable.

Variable	Factor loading	CR	AVE	Cronbach’sα
HAIC	0.775 ~ 0.797	0.919	0.619	0.919
CA	0.736 ~ 0.806	0.876	0.586	0.876
HA	0.759 ~ 0.788	0.882	0.599	0.882
AIL	0.773 ~ 0.794	0.904	0.610	0.903
DC	0.724 ~ 0.796	0.874	0.582	0.874

#### Pearson correlation.

To investigate preliminary bivariate relationships and diagnose potential multicollinearity, this study conducted Pearson correlation analyses on the core variables; the results are presented in Table 9.

**Table 9:** Descriptive statistics and correlation coefficients.

	1	2	3	4	5	6	7	8	9	10	11	12
Gender (1)	1											
Age (2)	0.016	1										
Education (3)	−0.06	0.230**	1									
Major (4)	0.051	−0.093*	−0.003	1								
Experience (5)	0.014	−0.079	−0.022	−0.042	1							
Frequency of AI Use (6)	0.075	0.032	0.021	0.015	0.281**	1						
AI Design Courses Taken(7)	−0.031	0.010	−0.045	−0.065	−0.010	−0.086	1					
HAIC(8)	0.047	0.032	−0.021	0.015	0.078	0.101*	−0.048	1				
CA(9)	0.042	0.005	−0.081	0.052	0.031	0.088	0.032	0.298**	1			
HA(10)	−0.01	−0.103*	0.094*	0.045	0.006	0.011	−0.042	0.260**	−0.003	1		
AIL(11)	0.005	−0.061	0.104*	−0.037	−0.006	−0.005	0.053	−0.057	0.021	0.132**	1	
DC(12)	0.075	0.053	0.018	0.018	0.102*	0.07	−0.044	0.312**	0.435**	−0.159**	−0.019	1
Average	1.592	2.731	1.284	2.783	3.099	2.029	0.188	4.225	4.463	4.148	4.173	4.409
Standard Deviation	0.492	0.79	0.569	1.411	1.104	0.714	0.391	1.42	1.399	1.426	1.414	1.408

*p < 0.05**p < 0.01

Regarding descriptive statistics, the distributions of the control variables aligned with expectations: the mean for gender was 1.592, indicating a relatively balanced gender ratio; the mean for age was 2.731 (SD = 0.790), suggesting that respondents were primarily young adults; AI usage frequency (M = 2.029, SD = 0.714) and usage duration (M = 3.099, SD = 1.104) indicated that participants engaged with AI tools at a moderate-to-low intensity; notably, only 18.84% of the sample had received formal AI design education. Concerning core variables, the mean score for HAIC was 4.225; both CA (M = 4.463) and DC (M = 4.409) were at relatively high levels, while the mean HA score of 4.148 also warranted attention.

In terms of correlational patterns, HAIC was significantly positively correlated with DC (r = 0.312, p < 0.01), providing preliminary support for the main hypothesis. Regarding the dual pathways of stress appraisal: HAIC was significantly positively correlated with CA (r = 0.298, p < 0.01), suggesting that deeper human-AI collaboration is more likely to be perceived as a growth-oriented stressor; concurrently, HAIC was significantly positively correlated with HA (r = 0.260, p < 0.01), implying that high-intensity collaboration may also trigger concerns regarding creative autonomy or skill substitution. Notably, CA exhibited a strong positive correlation with DC (r = 0.435, p < 0.01), whereas HA showed a significant negative correlation with DC (r = −0.159, p < 0.01). This stark contrast elucidates the divergent mechanisms through which challenge and hindrance appraisals influence creative development.

Furthermore, the correlation between CA and HA was non-significant (r = −0.003, p > 0.05), confirming that these two constructs represent independent dimensions, which is consistent with the theoretical tenets of Stress Appraisal Theory. Among the control variables, educational level was positively correlated with AIL (r = 0.104, p < 0.05) but also with HA (r = 0.094, p < 0.05); age was negatively correlated with HA (r = −0.103, p < 0.05), indicating that older students tend to perceive fewer hindrances. Crucially, the absolute values of all correlation coefficients were substantially below 0.50, far under the critical threshold of 0.70, suggesting the absence of serious multicollinearity and confirming the suitability of the data for subsequent regression and SEM analyses.

This study employed the Variance Inflation Factor (VIF) to assess the severity of multicollinearity. It is generally accepted that no serious multicollinearity exists when VIF < 5 (lenient criterion: < 10) and Tolerance > 0.2 (lenient criterion: > 0.1). As shown in Table 10, the VIF values for all independent variables ranged from 1.03 to 1.38, well below the threshold of 5; tolerance values ranged from 0.73 to 0.97, all above 0.2. These results indicate no significant multicollinearity among the five core variables, confirming that the regression coefficient estimates are stable and reliable.

**Table 10:** Multicollinearity diagnostics (VIF and tolerance).

Variable	Tolerance	VIF
HAIC	0.7703	1.2981
CA	0.7799	1.2821
HA	0.8472	1.1803
AIL	0.9702	1.0307
DC	0.7260	1.3774

### Hypothesis testing

#### Main effects and mediating effects.

After controlling for demographic variables (gender, age, education, major, AI usage duration, frequency, and AI courses), hierarchical regression analysis was conducted to test the hypotheses. The results are summarized in Table 11.

**Table 11:** Results of the regression analysis.

Variable	DC	CA		HA		DC
	Model 1	Model2	Model 3	Model 4	Model 5	Model 6
Constants	2.186**	2.968**	2.790**	3.370**	0.823**	1.878**
HAIC	0.298**	0.283**	0.282**	0.271**	0.746**	0.257**
CA						0.363**
HA						−0.229**
AIL			0.051		0.620**	
HAIC*AIL			−0.001		−0.112**	
Gender	0.164	0.050	0.048	−0.039	−0.032	0.137
Age	0.083	0.026	0.034	−0.253**	−0.229**	0.015
Education	0.044	−0.187	−0.202	0.326**	0.254*	0.187
Major	0.017	0.050	0.051	0.027	0.023	0.005
Experience	0.101	−0.010	−0.010	−0.025	−0.057	0.099
Frequency of AI Use	0.018	0.125	0.125	−0.019	0.018	−0.032
AI Design Courses	−0.088	0.184	0.175	−0.078	−0.109	−0.173
R2	0.110	0.103	0.105	0.098	0.164	0.287
Adjustment R2	0.096	0.088	0.086	0.083	0.147	0.274
*F* value	F(8,474)=7.395,p < 0.001	F(8,474)=6.821,p < 0.001	F(10,472)=5.559,p < 0.001	F(8,474)=6.469,p < 0.001	F(10,472)=9.289,p < 0.001	F(10,472)=19.222,p < 0.001

*p < 0.05,**p < 0.01

First, the direct effect of HAIC on DC was examined. Model 1 indicates that HAIC had a significant positive effect on DC (β = 0.298, p < 0.01), providing preliminary support for the main effect. Subsequently, the effects of HAIC on the two mediators were tested. Model 2 shows that HAIC significantly and positively predicted CA (β = 0.283, p < 0.01), supporting H1a. Model 4 demonstrates that HAIC significantly and positively predicted HA (β = 0.271, p < 0.01), supporting H1b.

To test the mediating roles, CA and HA were incorporated into Model 1 to construct Model 6. The results reveal that CA had a significant positive effect on DC (β = 0.363, p < 0.01), confirming H2a; meanwhile, HA had a significant negative effect on DC (β = −0.229, p < 0.01), confirming H2b. Notably, the direct effect of HAIC on DC remained significant (β = 0.257, p < 0.01), although the coefficient decreased from 0.298 to 0.257. This reduction indicates that CA and HA partially mediate the relationship between HAIC and DC, thereby supporting H3a and H3b. Furthermore, the R² of Model 6 increased from 0.110 to 0.287, signifying a substantial improvement in explanatory power.

#### Moderating effects.

AIL and its interaction term with HAIC were introduced into Models 2 and 4, respectively. Model 3 shows that the interaction term (HAIC×AIL) did not significantly predict CA (β = −0.001, p > 0.05); therefore, H4a was not supported. Conversely, Model 5 indicates that the interaction term significantly and negatively predicted HA (β = −0.112, p < 0.01), supporting H4b. This suggests that higher AIL attenuates the positive relationship between HAIC and HA.

To visually illustrate the moderating effect, a simple slope analysis was conducted by dividing the sample into low- and high-AIL groups based on one standard deviation above and below the mean (As shown in Fig 2).

**Fig 2:**
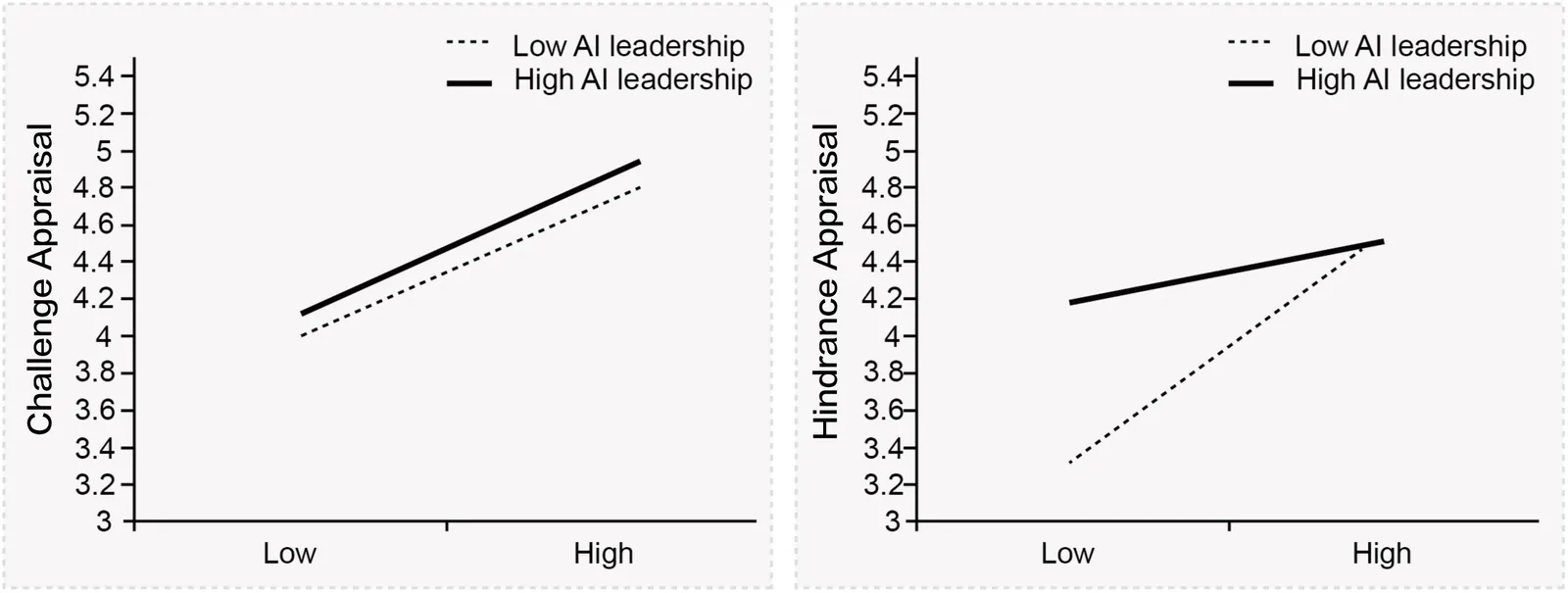
Simple slope analysis chart.

CA Path (H4a): Under both low and high AIL conditions, HAIC positively influenced CA. The regression slopes were nearly parallel with comparable magnitudes; although the slope was slightly steeper in the high AIL group, the difference was not statistically significant. This confirms that AIL does not exert a significant moderating effect on the HAIC → CA path, leading to the rejection of H4a.

HA Path (H4b): Under low AIL conditions, the regression slope was steeper, indicating a stronger positive effect of HAIC on HA. Under high AIL conditions, the slope flattened considerably, indicating a significant attenuation of this positive effect. Notably, at low levels of HAIC, the HA level in the high AIL group was slightly higher; however, as HAIC increased, this gap narrowed and eventually converged. These findings robustly support H4b, indicating that AIL acts as a buffer against the hindrance appraisal induced by HAIC.

### Testing for mediating effects

To further ascertain the robustness of the mediating effects, a bias-corrected percentile bootstrap method with 5,000 resamples was employed to test the indirect pathways. The results are presented in Table 12.

**Table 12:** Results of the bootstrap test for mediating effects.

Impact Effects	Path	Effect	BootSE	BootLLCI	BootULCI
Overall Effect	HAIC→DC	0.298	0.043	0.213	0.383
Direct Effect	HAIC→DC	0.257	0.042	0.174	0.341
Indirect Effect	HAIC→CA → DC	0.103	0.028	0.053	0.162
	HAIC→HA → DC	−0.062	0.02	−0.105	−0.026

The total effect of HAIC on DC was significant (Effect = 0.298, 95% CI = [0.213, 0.383]). After controlling for the mediating variables, the direct effect remained significant (Effect = 0.257, 95% CI = [0.174, 0.341]), confirming that CA and HA partially mediate the relationship. Both indirect effects were statistically significant: the indirect effect of the HAIC → CA → DC path was 0.103 (95% CI = [0.053, 0.162]), indicating a positive mediating effect and supporting H3a; the indirect effect of the HAIC → HA → DC path was −0.062 (95% CI = [−0.105, −0.026]), representing a negative mediating effect and supporting H3b. Crucially, none of the aforementioned confidence intervals included zero, validating the presence of these mediation pathways. These findings indicate that HAIC not only directly promotes DC but also operates through a dual mediation mechanism: it generates a positive indirect effect by enhancing CA, while simultaneously exerting a negative indirect effect by intensifying HA, thereby forming a “double-edged sword” mediation model.

### Test of moderated mediation effects

To further examine the moderating role of AIL on the indirect effects of the two mediation paths, a bias-corrected percentile bootstrap method with 5,000 resamples was employed; the results are presented in Table 13.

**Table 13:** Results of the moderated mediating effect test.

Mediating variable	Level	Effect	BootSE	BootLLCI	BootULCI
CA	Low level(−1SD)	0.103	0.037	0.041	0.186
	Average	0.104	0.029	0.053	0.167
	High-level(+1SD)	0.104	0.032	0.047	0.173
HA	Low level(−1SD)	−0.100	0.027	−0.158	−0.050
	Average	−0.063	0.020	−0.108	−0.029
	High-level(+1SD)	−0.027	0.020	−0.072	0.007
Index of moderated mediation	CA (HAIC→CA → DC)	−0.0004	0.0131	−0.0275	0.0244
	HA (HAIC→HA → DC)	0.0255	0.0087	0.0119	0.0471

Challenge Pathway (H5a):At low (−1 SD), mean, and high (+1 SD) levels of AIL, the indirect effects mediated by CA were 0.103 (95% CI = [0.041, 0.186]), 0.104 (95% CI = [0.053, 0.167]), and 0.104 (95% CI = [0.047, 0.173]), respectively. All confidence intervals excluded zero, indicating significant indirect effects. However, the effect sizes were nearly identical across the three conditions, suggesting that AIL did not significantly alter the strength of this indirect pathway. Consequently, H5a was not supported.

Hindrance Pathway (H5b): At low levels of AIL, the indirect effect via HA was −0.100 (95% CI = [−0.158, −0.050]); at mean levels, it was −0.063 (95% CI = [−0.108, −0.029]); both were statistically significant. Crucially, at high levels of AIL, this negative indirect effect attenuated to −0.027 (95% CI = [−0.072, 0.007]), with the confidence interval now including zero, rendering the effect non-significant. This pattern indicates that higher AIL significantly weakens the negative indirect effect of HAIC on DC through HA, thereby supporting H5b.

To formally examine whether the conditional indirect effects differed significantly as a function of perceived teacher AIL, we computed the index of moderated mediation for each mediation pathway, using the same bias-corrected percentile bootstrap procedure with 5,000 resamples (PROCESS Model 7; first-stage moderation). As shown in Table 13, the index of moderated mediation for the HAIC → HA → DC pathway was 0.0255 (Boot SE = 0.0087, 95% CI = [0.0119, 0.0471]). Because the confidence interval excluded zero, the negative indirect effect of HAIC on DC via hindrance appraisal was significantly attenuated at higher levels of perceived teacher AIL, providing formal statistical evidence for H5b and corroborating the simple-slope and conditional indirect effect analyses reported above. In contrast, the index of moderated mediation for the HAIC → CA → DC pathway was −0.0004 (Boot SE = 0.0131, 95% CI = [−0.0275, 0.0244]); because the confidence interval included zero, the positive indirect effect via challenge appraisal did not vary significantly across AIL levels, and H5a was therefore not supported. Taken together, the index-based tests confirm the asymmetric moderating pattern of perceived teacher AIL: it significantly buffers the inhibitory (hindrance-appraisal) pathway while exerting no significant influence on the facilitative (challenge-appraisal) pathway.

## Discussion

Grounded in Stress Appraisal Theory, this study constructed a moderated dual-mediation model to investigate the “double-edged sword” effect of HAIC on design students’ DC and to examine the boundary role of instructors’ AIL. The key findings are summarized as follows:

### The double-edged sword mechanism of HAIC on DC

The core finding confirms that the relationship between HAIC and DC is not a simple linear promotion but manifests as a significant “double-edged sword” effect, driven by differentiated psychological appraisal mechanisms. This supports the dual-path hypothesis derived from Stress Appraisal Theory.

First, the facilitative pathway (CA) was empirically validated. Results indicate that HAIC significantly and positively influenced CA (β = 0.283, p < 0.01), which in turn had a significant positive effect on DC (β = 0.363, p < 0.01). Bootstrap tests further confirmed a significant positive mediating effect of CA (indirect effect = 0.103, 95% CI = [0.053, 0.162]), aligning with prior research [42,46,48]. This suggests that when students perceive AI as a controllable tool and a growth opportunity, HAIC functions primarily as a job resource. In this scenario, AI not only lowers technical barriers [4] but also stimulates curiosity and deep learning motivation, prompting students to logically deconstruct and critically reconstruct AI outputs, thereby achieving gains in technical skills and creative abilities.

Second, the inhibitory pathway (HA) was also significant. Data analysis revealed that HAIC had a significant positive effect on HA (β = 0.271, p < 0.01), while HA significantly hindered DC (β = −0.229, p < 0.01). Mediation analysis supported a significant negative indirect effect via HA (indirect effect = −0.062, 95% CI = [−0.105, −0.026]), corroborating the applicability of Cognitive Offloading Theory [13] in design education. When AI-induced cognitive load, uncertainty, and ethical risks exceed students’coping thresholds, they trigger defensive dependence and anxiety [61], leading to a decline in critical thinking and originality, thereby inhibiting DC.

Notably, a comparison of the path coefficients revealed that the facilitating effect of CA (positive) outweighed the inhibitory effect of HA (negative) in absolute terms. This implies that, within the design discipline, the current “sense of empowerment” derived from AI outweighs the “sense of deprivation,” providing an optimistic empirical basis for human-AI collaborative education models. Nevertheless, the significant presence of the hindrance pathway underscores that potential negative impacts must not be overlooked.

### The asymmetric contingency mechanism of AIL

This study uncovers a critical asymmetric contingency effect of AIL on the HAIC-DC relationship. Results indicate that AIL acts as a significant negative buffer on the HAIC → HA path (H4b supported, β = −0.112, p < 0.01), effectively insulating students from the psychological burdens induced by technology. However, AIL failed to significantly augment the HAIC → CA pathway (H4a rejected, β = 0.001, p > 0.05). This null finding, while unexpected, may reflect the unique contextual and cognitive specificities of design education, rather than a failure of the hypothesized mechanism per se. It should be acknowledged that the following accounts are post hoc and exploratory in nature, representing potential explanations rather than definitive conclusions. Grounded in the idiosyncrasies of design cognition and the current state of educational practice, we tentatively propose four potential explanations:

### Alignment with the non-linear nature of design cognition

Unlike the acquisition of general vocational skills, design innovation is inherently non-linear, often emerging from spontaneous inspiration and heuristic exploration [62]. The core of DC creative ideation is rooted in endogenous motivational cycles of divergence, convergence, and reflection. During the divergent phase of ideation, cognitive processing is highly tacit and non-linear [63], which may impose a natural ceiling on the extent to which external instructional leadership can directly intervene to foster CA. In contrast, during the convergent phase (technical execution and ethical scrutiny), AIL provides essential scaffolding that mitigates cognitive load and HA. Therefore, the non-significant moderating effect on the CA pathway might be attributed to the limited penetrability of external guidance into the early, intuitive stages of creative cognition.

### Functional reorientation: From catalyst to risk steward

As AI transitions into an “intelligent collaborator” [64], the pedagogical role of instructors appears to be shifting organically from that of an inspirational catalyst to a cognitive risk steward [65]. In this evolving context, the utility of AIL may lie primarily in establishing a secure, ethical, and structured infrastructure for collaboration, rather than usurping students’ creative agency to stimulate breakthrough ideas. This delineation potentially explains why AIL demonstrates robust efficacy in “error correction” and “risk mitigation” (suppressing HA), yet exhibits limited capacity to artificially amplify “challenge appraisal” (enhancing CA), which is intrinsically tied to individual creative drive.

### Potential constraints of structural lags in faculty development

It is also plausible that the current manifestation of AIL is constrained by systemic developmental bottlenecks. The data reveal a severe supply-demand gap: only 18.84% of students received systematic AI training, suggesting that the majority of educators remain in transition from “traditional design instructors” to “AI-collaborative facilitators.” We speculate that this structural lag in faculty upskilling wherein teachers have not yet fully integrated AI tooling, ethics, and collaborative protocols into the curriculum may temporarily limit the overall potency of AIL. Consequently, the full potential of AIL to elevate the positive pathway might be underdeveloped in the current educational landscape, representing a boundary condition that warrants further investigation in contexts with more mature AI pedagogy.

### A potential ceiling effect of challenge appraisal

Finally, a potential ceiling‑effect of challenge appraisal may also account for the non‑significant moderation. Design students may already perceive HAIC as a growth‑oriented challenge based on their intrinsic creative motivation and prior design experience. Accordingly, teacher AI‑leadership interventions may offer limited additional gains for further elevating challenge appraisal. This interpretation also awaits future empirical verification.

## Conclusion

### Theoretical contributions

Compared to extant literature, this study makes the following significant theoretical contributions:

(1) Unraveling the “Black Box” of HAIC’s Influence on DC. Existing research often isolates the empowerment or risks of AI [5,15], lacking a dialectical integration mechanism. By introducing Stress Appraisal Theory into design education, this study confirms that HAIC possesses dual attributes of “gain” and “loss.” Crucially, it is the first to elucidate why the use of identical AI tools leads to heterogeneous competency development outcomes, achieved through the dual mediating mechanisms of Challenge and Hindrance Appraisal.
(2) Identifying the asymmetric boundary conditions of AI leadership. Prior AI‑collaboration research largely prioritizes student‑individual factors such as AI literacy [44], while contextual instructor‑level moderators remain under‑studied. This study identifies an asymmetric moderating pattern: AI leadership significantly buffers the harmful hindrance‑appraisal pathway, yet fails to amplify the beneficial challenge‑appraisal pathway. This pattern stands in contrast to findings from general corporate‑workplace digital‑leadership literature, where leadership is commonly expected to amplify positive motivational and innovative outcomes alongside mitigating risks [37,48]. Our results suggest that within design‑education contexts, educators’ AI‑leadership function may center more upon the role of “cognitive risk steward” to protect students from technology‑related psychological costs, rather than acting primarily as an innovation catalyst to boost creative challenge appraisals. This distinction reveals context‑specific mechanisms for AI leadership within pedagogical settings that cannot be directly extrapolated from corporate‑sector leadership theories.
(3) Extending Stress Appraisal Theory to Generative AI Contexts. This study validates that students’ perceptions of AI are not binary but involve coexisting evaluations of challenges and threats. This finding enriches the explanatory power of Lazarus and Folkman’s (1984) [25] seminal theory when applied to generative AI as a novel technological stressor.

### Implications for practice

A striking finding from the descriptive statistics is that only 18.84% of participating students had taken formal AI design courses. This stark gap between the widespread adoption of AI tools and the lack of systematic AI pedagogy underscores the urgent need for faculty development programs that equip teachers with AI leadership competencies.

Specifically, we recommend incorporating ‘AI critique’ or ‘human-AI collaboration reflection’ as a mandatory component in student portfolio assessments. This would require students to critically evaluate AI-generated outputs and document their iterative decision-making process, thereby mitigating the risk of creative homogenization while fostering the metacognitive skills central to competence development.

Based on these findings, the following recommendations are proposed for design institutions and educators:

(1) Transform the Teacher’s Role from “Skill Transmitter” to “Cognitive Risk Manager.” Given that AIL failed to significantly stimulate CA (H4a unsupported), teachers should prioritize cognitive correction over mere skill transfer. Pedagogical practice should emphasize AI ethics, copyright discernment, and critical thinking, while clearly defining the boundaries of human-AI collaboration. Guiding students to critically scrutinize AI outputs can prevent originality depletion and cognitive decline caused by over-reliance, ensuring technology serves design innovation.
(2) Systematically Enhance Teachers’ AIL. With only 18.84% of students having received formal AI training, institutions must urgently implement foundational faculty development programs. Training should move beyond superficial tool usage and focus on systematic upskilling in prompt engineering, AI-augmented instructional design, and algorithmic ethics, transforming instructors from “tool users” into “curricular integrators.”
(3) Establish a Low-Friction, High-Empowerment Technical Ecosystem. In response to the null effect of H4a, institutions should avoid coercing students to “embrace AI” through assessment pressure. Instead, efforts should focus on optimizing hardware/software infrastructure and reducing technological friction (e.g., providing computing power support), allowing students to naturally perceive AI as a valuable assistant.
(4) Optimize the design‑competence evaluation system. Prior experimental work demonstrates that AI‑assisted design workflows may produce design fixation and creative homogenization among student designers [16]. Accordingly, assessment criteria should reduce over‑reliance on aesthetic scores for final AI‑generated visual outputs. Instead, evaluation should prioritize students’ documented reasoning behind proposal selection, iterative decision‑making trails, and depth of humanistic‑need consideration throughout the design process. As a concrete curriculum measure, educators can mandate “AI critique” or human‑AI‑collaboration reflective statements as a compulsory component of student design portfolios. Students would be required to critically evaluate strengths, limitations, biases, and ethical risks within AI‑generated outputs and document how they intervene, modify, and reinterpret AI suggestions. This portfolio requirement targets both hindrance‑appraisal risk factors and helps foster the critical‑thinking capacity central to design competence development.

### Research limitations and future directions

This study has several limitations that warrant future refinement:

First, regarding methodological constraints, the use of cross-sectional data precludes definitive causal inferences. Future research should employ multi-wave longitudinal designs or experimental methods to verify the long-term dynamic effects of HAIC on DC. Furthermore, this study adopted convenience sampling, recruiting participants solely from East China (Jiangsu, Zhejiang, and Shanghai). Regional disparities in AI education resources, policy implementation, and tool accessibility may constrain the external validity and generalizability of our findings. Future research should adopt multi-region or cross-cultural sampling to replicate and extend the present model.

Second, concerning theoretical scope, the focus on a specific disciplinary context (design majors) limits the breadth of the model. Future studies should extend this model to other fields (e.g., engineering, humanities) to conduct comparative analyses and test the model’s boundary conditions.

Finally, at the individual level, this study primarily examined AIL at the instructor level. Future research could incorporate individual-level moderators such as students’ AI literacy or personality traits (e.g., the Big Five) to explore heterogeneous responses to the double-edged sword effect.

## Supporting information

S1 File Raw data. (XLSX)
